# Supplementary material for: Efficient and Informative Laboratory Testing for Rapid Confirmation of H5N1 (Clade 2.3.4.4) High-Pathogenicity Avian Influenza Outbreaks in the United Kingdom
Source: Viruses. 2023 Jun 9;15(6):1344. doi: 10.3390/v15061344 (PMC10304448; doi:10.3390/v15061344)
Supplement: Supplementary file 1 [file viruses-15-01344-s001.zip › Fig S1.pptx]

## Slide 1
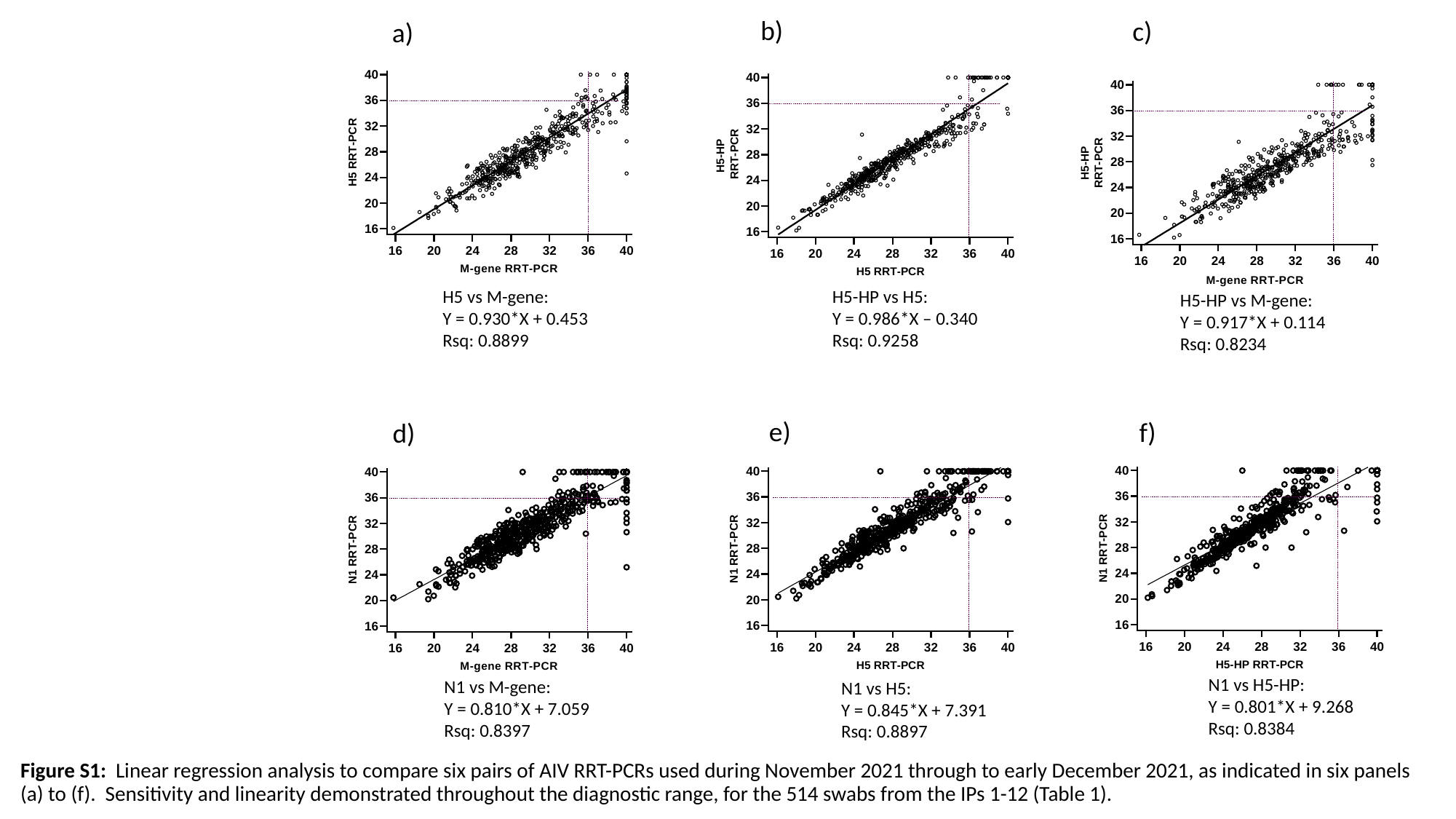

b)
 c)
 a)
H5-HP vs H5:
Y = 0.986*X – 0.340
Rsq: 0.9258
H5 vs M-gene:
Y = 0.930*X + 0.453
Rsq: 0.8899
H5-HP vs M-gene:
Y = 0.917*X + 0.114
Rsq: 0.8234
 e)
 f)
 d)
N1 vs H5-HP:
Y = 0.801*X + 9.268
Rsq: 0.8384
N1 vs M-gene:
Y = 0.810*X + 7.059
Rsq: 0.8397
N1 vs H5:
Y = 0.845*X + 7.391
Rsq: 0.8897
Figure S1: Linear regression analysis to compare six pairs of AIV RRT-PCRs used during November 2021 through to early December 2021, as indicated in six panels (a) to (f). Sensitivity and linearity demonstrated throughout the diagnostic range, for the 514 swabs from the IPs 1-12 (Table 1).
