# Supplementary material for: Efficient and Informative Laboratory Testing for Rapid Confirmation of H5N1 (Clade 2.3.4.4) High-Pathogenicity Avian Influenza Outbreaks in the United Kingdom
Source: Viruses. 2023 Jun 9;15(6):1344. doi: 10.3390/v15061344 (PMC10304448; doi:10.3390/v15061344)
Supplement: Supplementary file 1 [file viruses-15-01344-s001.zip › Fig S2.pptx]

## Slide 1
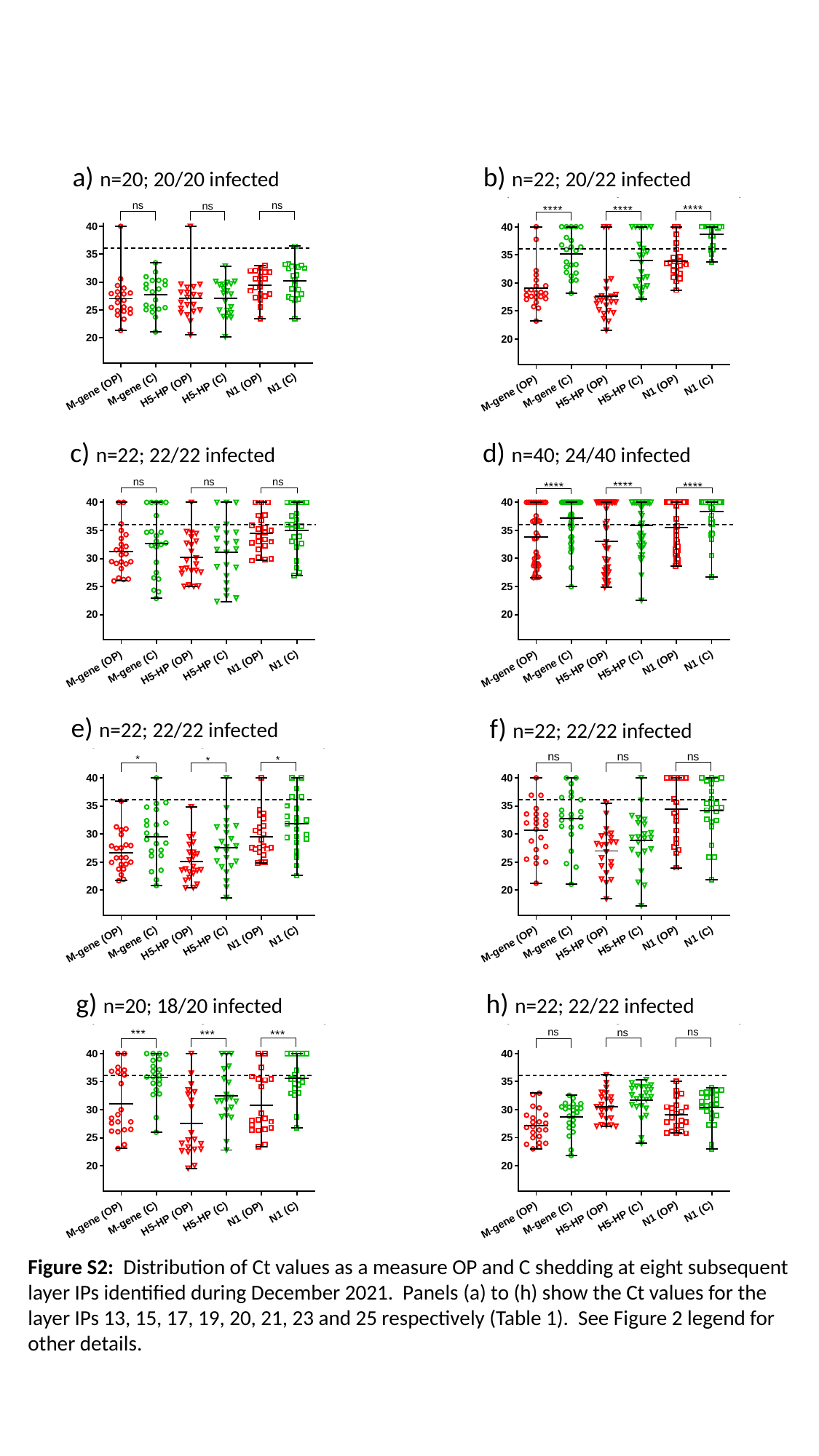

b) n=22; 20/22 infected
 a) n=20; 20/20 infected
 c) n=22; 22/22 infected
 d) n=40; 24/40 infected
 e) n=22; 22/22 infected
 f) n=22; 22/22 infected
 h) n=22; 22/22 infected
 g) n=20; 18/20 infected
Figure S2: Distribution of Ct values as a measure OP and C shedding at eight subsequent layer IPs identified during December 2021. Panels (a) to (h) show the Ct values for the layer IPs 13, 15, 17, 19, 20, 21, 23 and 25 respectively (Table 1). See Figure 2 legend for other details.
