# Supplementary material for: Efficient and Informative Laboratory Testing for Rapid Confirmation of H5N1 (Clade 2.3.4.4) High-Pathogenicity Avian Influenza Outbreaks in the United Kingdom
Source: Viruses. 2023 Jun 9;15(6):1344. doi: 10.3390/v15061344 (PMC10304448; doi:10.3390/v15061344)
Supplement: Supplementary file 1 [file viruses-15-01344-s001.zip › Fig S3.pptx]

## Slide 1
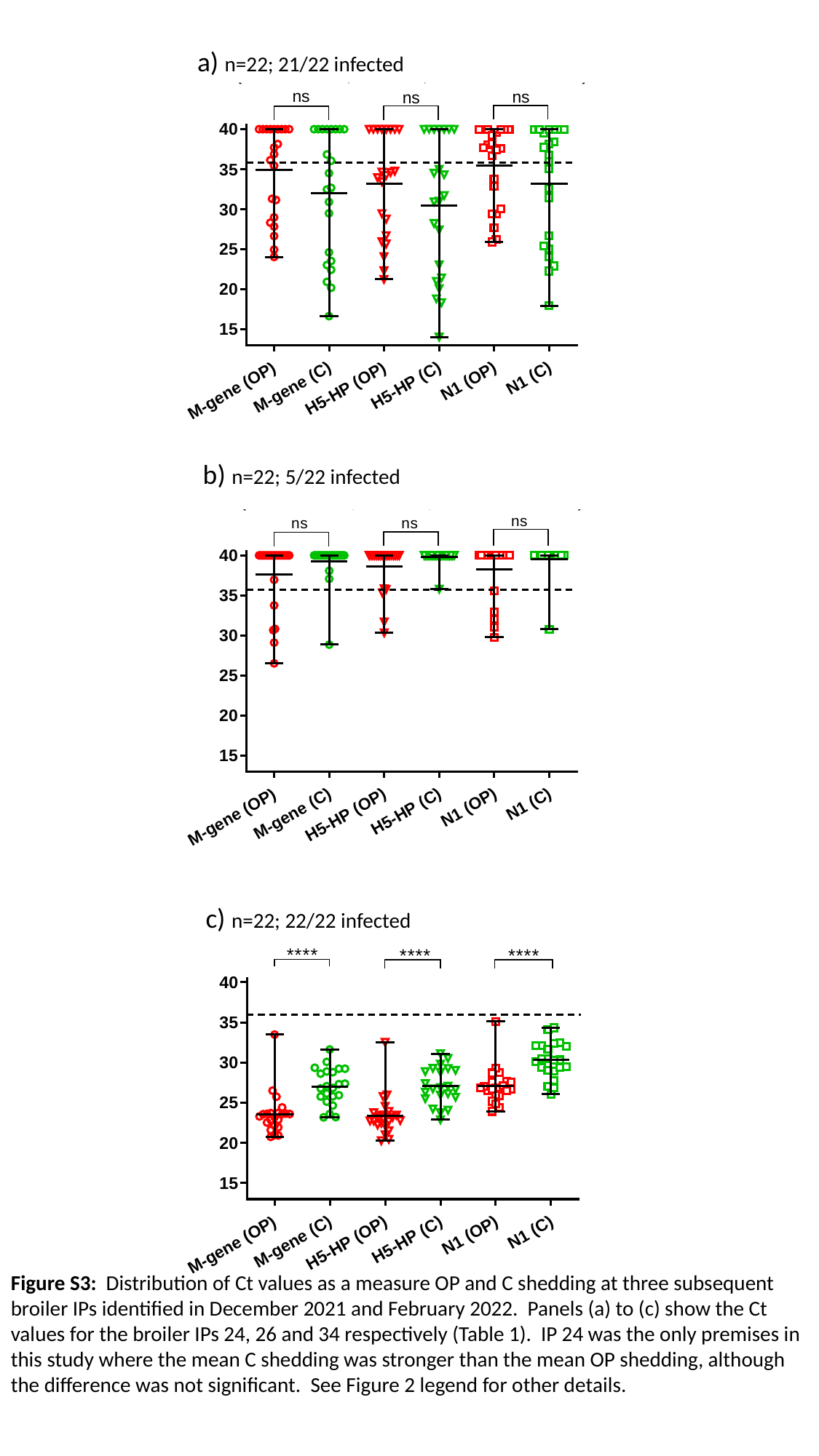

a) n=22; 21/22 infected
 b) n=22; 5/22 infected
 c) n=22; 22/22 infected
Figure S3: Distribution of Ct values as a measure OP and C shedding at three subsequent broiler IPs identified in December 2021 and February 2022. Panels (a) to (c) show the Ct values for the broiler IPs 24, 26 and 34 respectively (Table 1). IP 24 was the only premises in this study where the mean C shedding was stronger than the mean OP shedding, although the difference was not significant. See Figure 2 legend for other details.
