# Supplementary material for: Efficient and Informative Laboratory Testing for Rapid Confirmation of H5N1 (Clade 2.3.4.4) High-Pathogenicity Avian Influenza Outbreaks in the United Kingdom
Source: Viruses. 2023 Jun 9;15(6):1344. doi: 10.3390/v15061344 (PMC10304448; doi:10.3390/v15061344)
Supplement: Supplementary file 1 [file viruses-15-01344-s001.zip › Fig S4.pptx]

## Slide 1
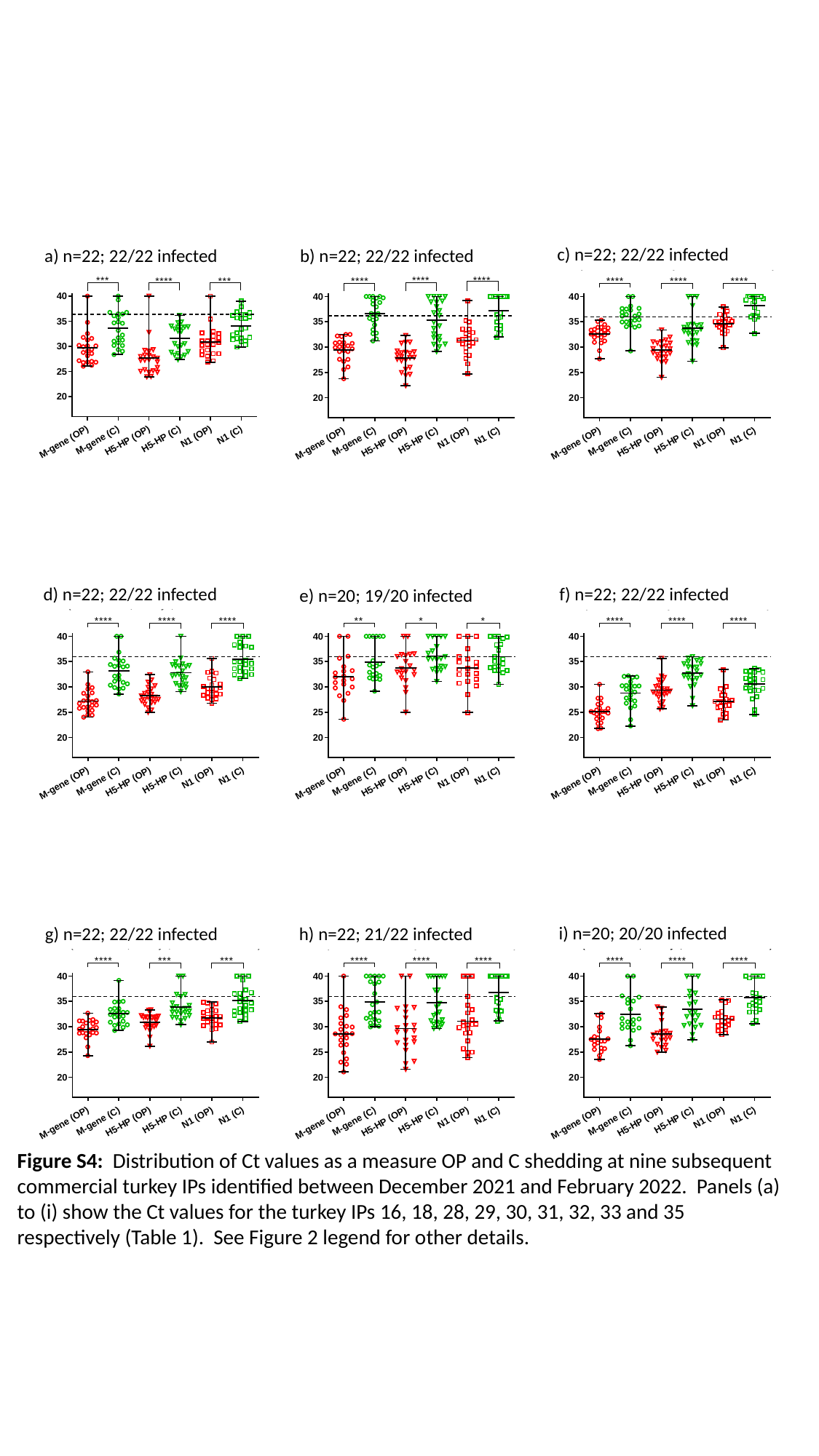

c) n=22; 22/22 infected
 a) n=22; 22/22 infected
 b) n=22; 22/22 infected
 d) n=22; 22/22 infected
 f) n=22; 22/22 infected
 e) n=20; 19/20 infected
 i) n=20; 20/20 infected
 g) n=22; 22/22 infected
 h) n=22; 21/22 infected
Figure S4: Distribution of Ct values as a measure OP and C shedding at nine subsequent commercial turkey IPs identified between December 2021 and February 2022. Panels (a) to (i) show the Ct values for the turkey IPs 16, 18, 28, 29, 30, 31, 32, 33 and 35 respectively (Table 1). See Figure 2 legend for other details.
