# Supplementary material for: Efficient and Informative Laboratory Testing for Rapid Confirmation of H5N1 (Clade 2.3.4.4) High-Pathogenicity Avian Influenza Outbreaks in the United Kingdom
Source: Viruses. 2023 Jun 9;15(6):1344. doi: 10.3390/v15061344 (PMC10304448; doi:10.3390/v15061344)
Supplement: Supplementary file 1 [file viruses-15-01344-s001.zip › Fig S5.pptx]

## Slide 1
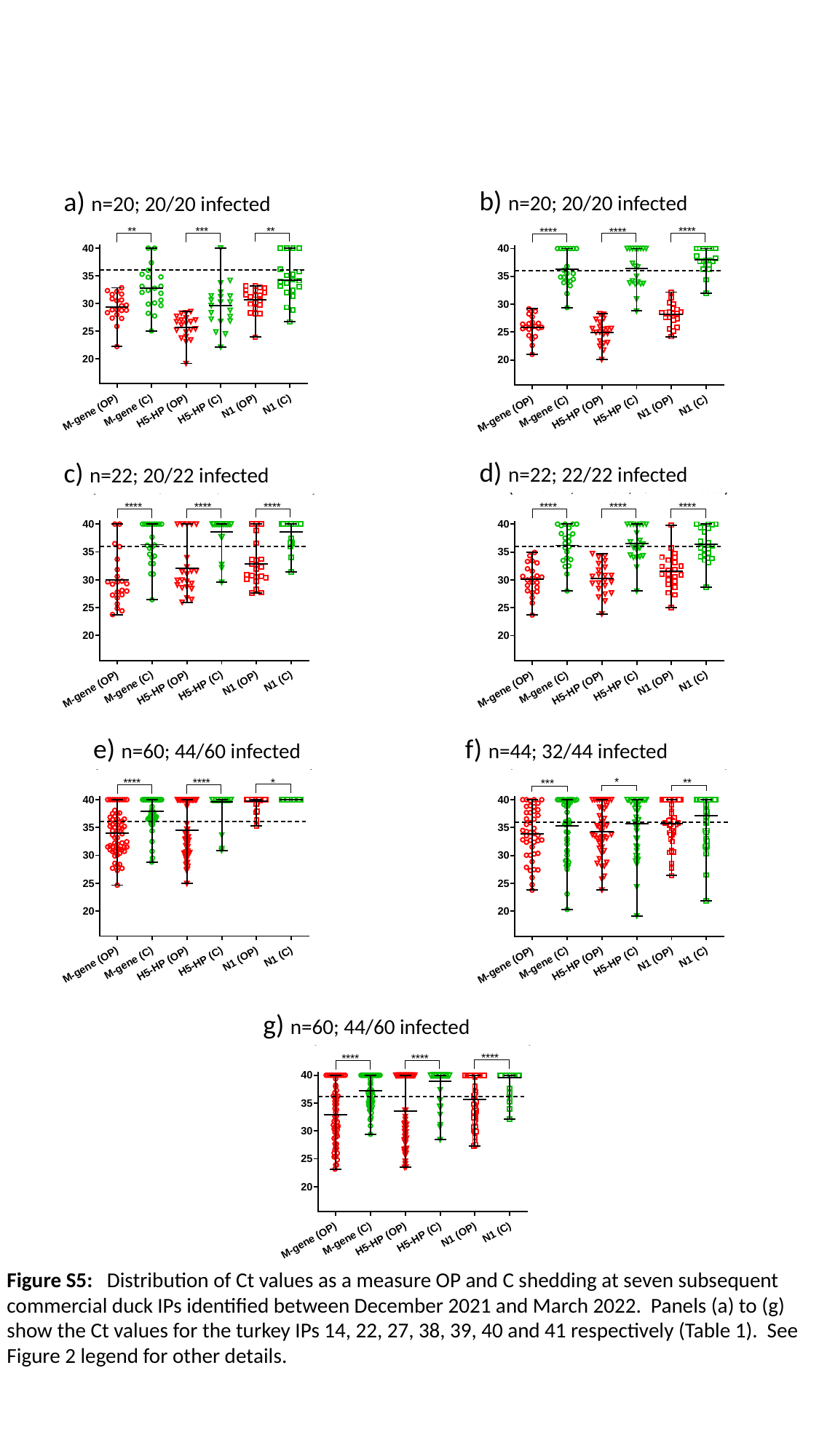

b) n=20; 20/20 infected
 a) n=20; 20/20 infected
 d) n=22; 22/22 infected
 c) n=22; 20/22 infected
 e) n=60; 44/60 infected
 f) n=44; 32/44 infected
g) n=60; 44/60 infected
Figure S5: Distribution of Ct values as a measure OP and C shedding at seven subsequent commercial duck IPs identified between December 2021 and March 2022. Panels (a) to (g) show the Ct values for the turkey IPs 14, 22, 27, 38, 39, 40 and 41 respectively (Table 1). See Figure 2 legend for other details.
