# Supplementary material for: Efficient and Informative Laboratory Testing for Rapid Confirmation of H5N1 (Clade 2.3.4.4) High-Pathogenicity Avian Influenza Outbreaks in the United Kingdom
Source: Viruses. 2023 Jun 9;15(6):1344. doi: 10.3390/v15061344 (PMC10304448; doi:10.3390/v15061344)
Supplement: Supplementary file 1 [file viruses-15-01344-s001.zip › Fig S6.pptx]

## Slide 1
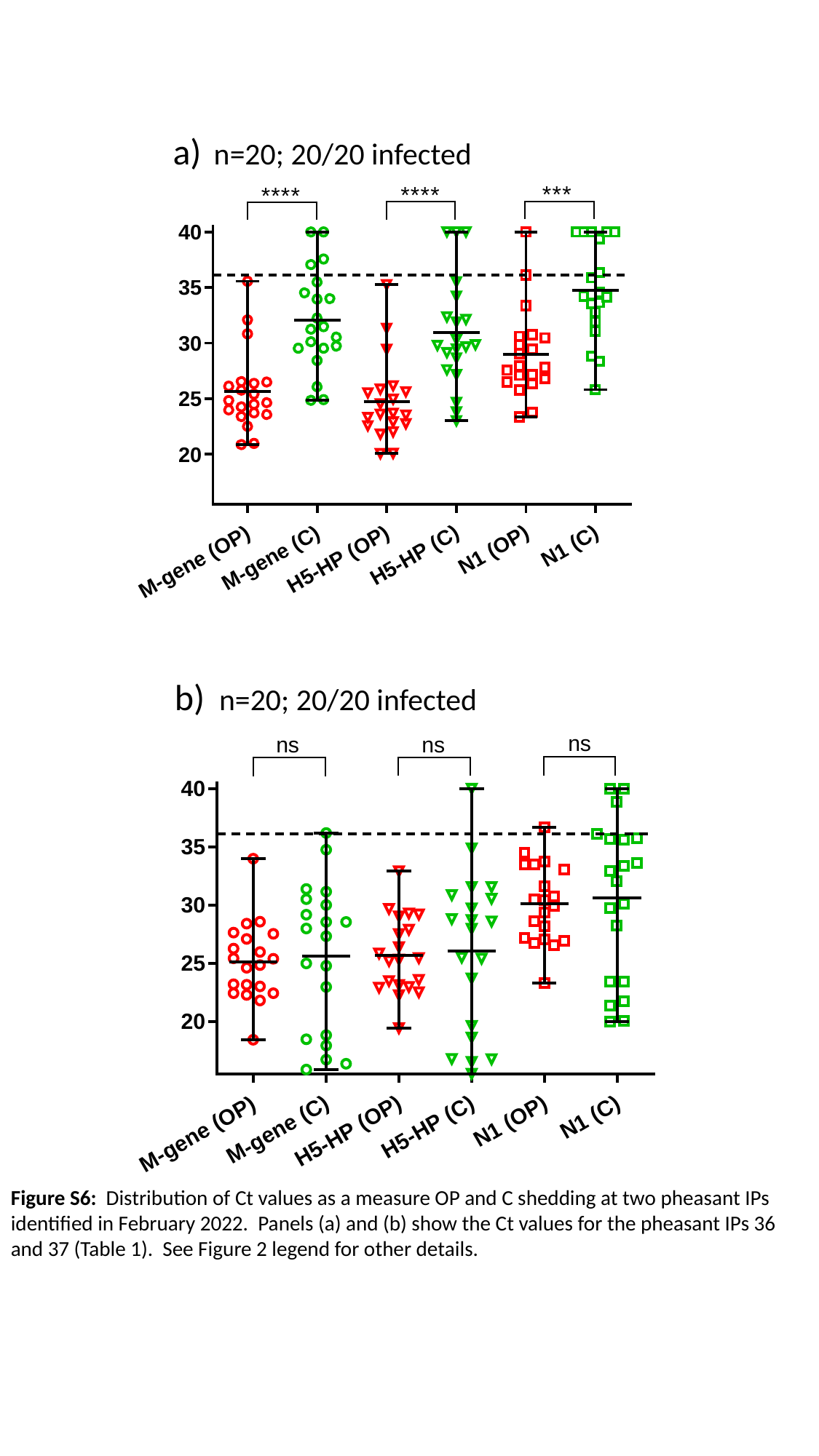

a) n=20; 20/20 infected
 b) n=20; 20/20 infected
Figure S6: Distribution of Ct values as a measure OP and C shedding at two pheasant IPs identified in February 2022. Panels (a) and (b) show the Ct values for the pheasant IPs 36 and 37 (Table 1). See Figure 2 legend for other details.
