# Supplementary material for: Efficient and Informative Laboratory Testing for Rapid Confirmation of H5N1 (Clade 2.3.4.4) High-Pathogenicity Avian Influenza Outbreaks in the United Kingdom
Source: Viruses. 2023 Jun 9;15(6):1344. doi: 10.3390/v15061344 (PMC10304448; doi:10.3390/v15061344)
Supplement: Supplementary file 1 [file viruses-15-01344-s001.zip › Fig S7.pptx]

## Slide 1
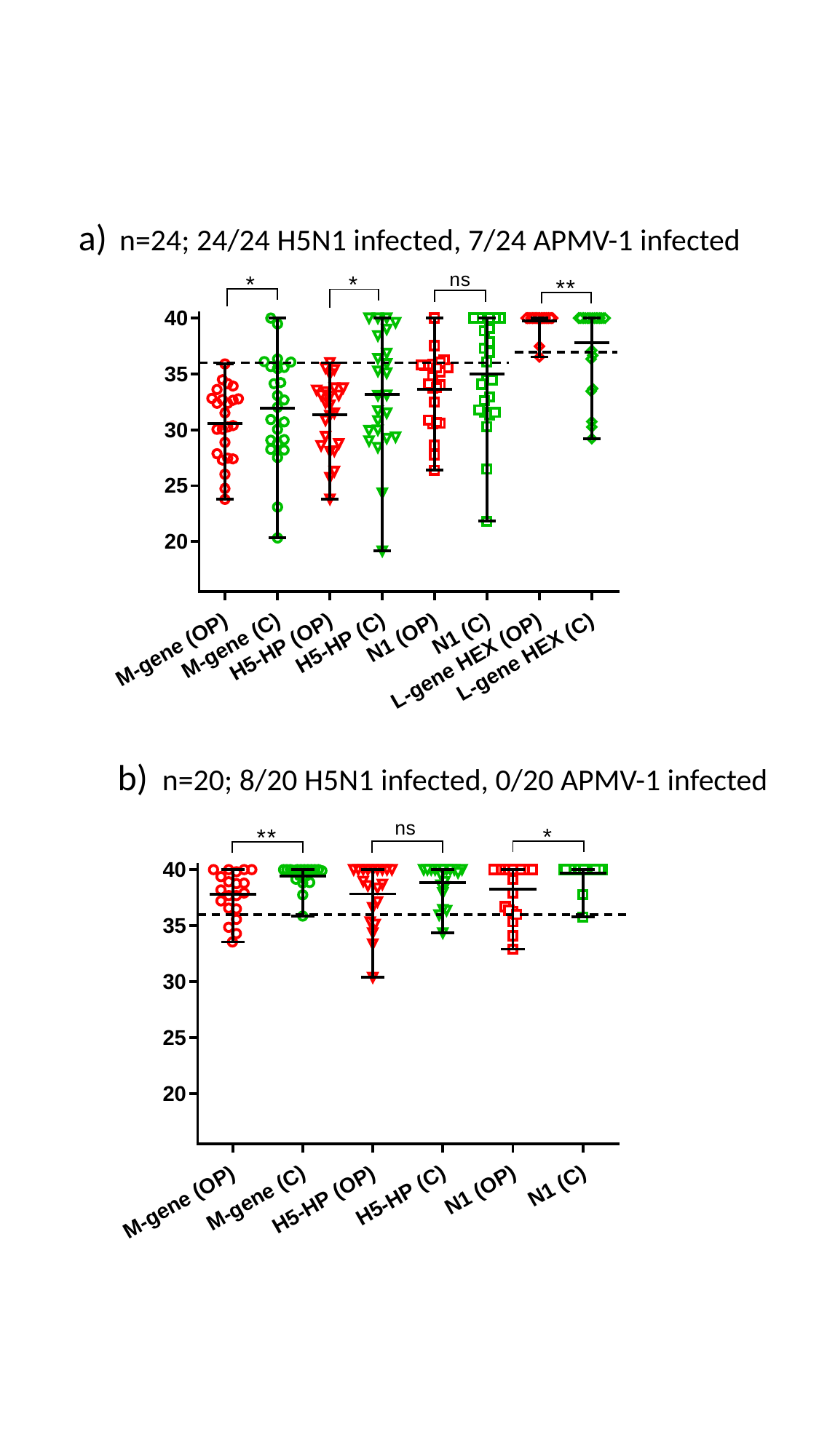

a) n=24; 24/24 H5N1 infected, 7/24 APMV-1 infected
b) n=20; 8/20 H5N1 infected, 0/20 APMV-1 infected

## Slide 2
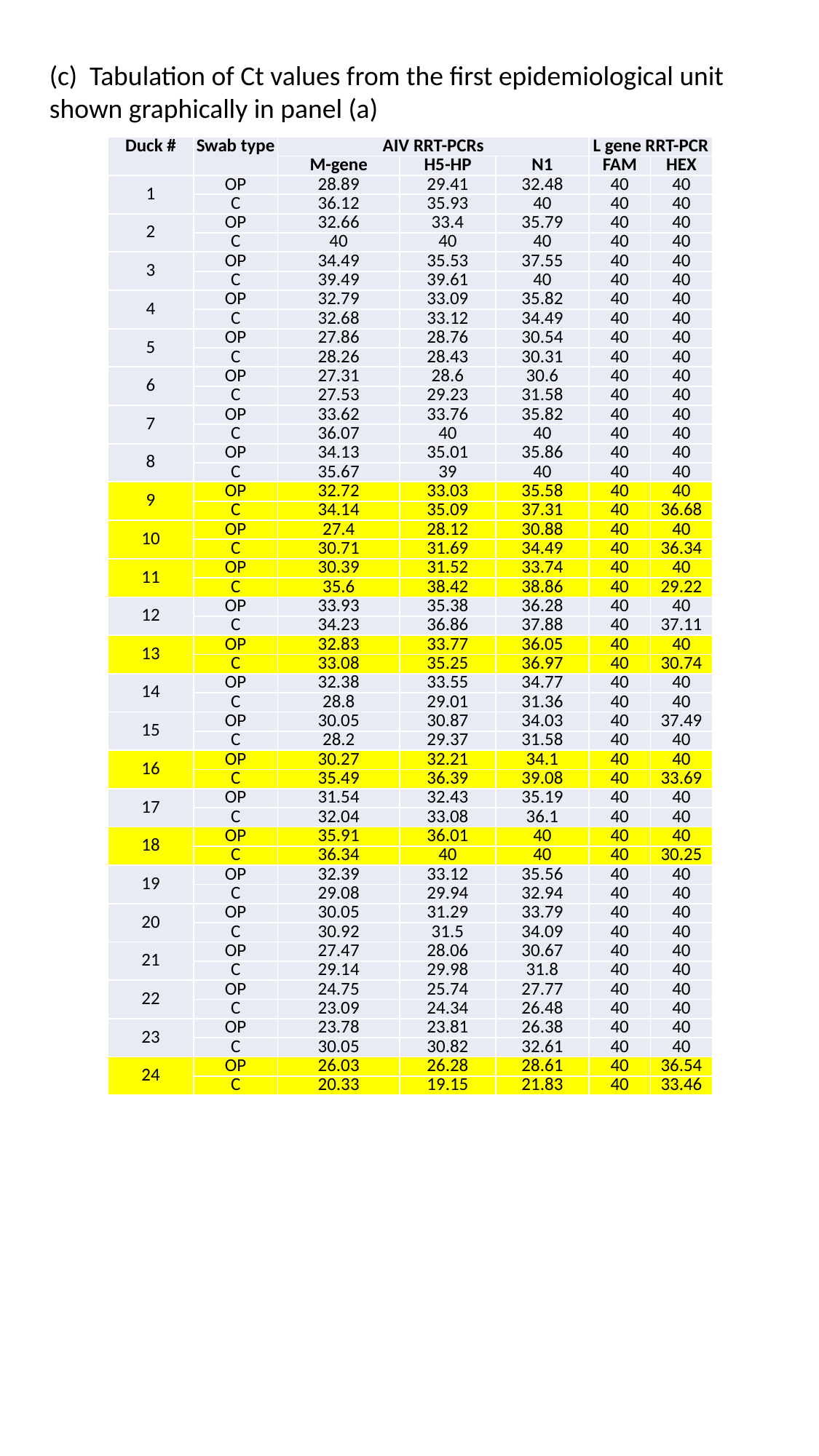

(c) Tabulation of Ct values from the first epidemiological unit shown graphically in panel (a)
| Duck # | Swab type | AIV RRT-PCRs | | | L gene RRT-PCR | |
| --- | --- | --- | --- | --- | --- | --- |
| | | M-gene | H5-HP | N1 | FAM | HEX |
| 1 | OP | 28.89 | 29.41 | 32.48 | 40 | 40 |
| | C | 36.12 | 35.93 | 40 | 40 | 40 |
| 2 | OP | 32.66 | 33.4 | 35.79 | 40 | 40 |
| | C | 40 | 40 | 40 | 40 | 40 |
| 3 | OP | 34.49 | 35.53 | 37.55 | 40 | 40 |
| | C | 39.49 | 39.61 | 40 | 40 | 40 |
| 4 | OP | 32.79 | 33.09 | 35.82 | 40 | 40 |
| | C | 32.68 | 33.12 | 34.49 | 40 | 40 |
| 5 | OP | 27.86 | 28.76 | 30.54 | 40 | 40 |
| | C | 28.26 | 28.43 | 30.31 | 40 | 40 |
| 6 | OP | 27.31 | 28.6 | 30.6 | 40 | 40 |
| | C | 27.53 | 29.23 | 31.58 | 40 | 40 |
| 7 | OP | 33.62 | 33.76 | 35.82 | 40 | 40 |
| | C | 36.07 | 40 | 40 | 40 | 40 |
| 8 | OP | 34.13 | 35.01 | 35.86 | 40 | 40 |
| | C | 35.67 | 39 | 40 | 40 | 40 |
| 9 | OP | 32.72 | 33.03 | 35.58 | 40 | 40 |
| | C | 34.14 | 35.09 | 37.31 | 40 | 36.68 |
| 10 | OP | 27.4 | 28.12 | 30.88 | 40 | 40 |
| | C | 30.71 | 31.69 | 34.49 | 40 | 36.34 |
| 11 | OP | 30.39 | 31.52 | 33.74 | 40 | 40 |
| | C | 35.6 | 38.42 | 38.86 | 40 | 29.22 |
| 12 | OP | 33.93 | 35.38 | 36.28 | 40 | 40 |
| | C | 34.23 | 36.86 | 37.88 | 40 | 37.11 |
| 13 | OP | 32.83 | 33.77 | 36.05 | 40 | 40 |
| | C | 33.08 | 35.25 | 36.97 | 40 | 30.74 |
| 14 | OP | 32.38 | 33.55 | 34.77 | 40 | 40 |
| | C | 28.8 | 29.01 | 31.36 | 40 | 40 |
| 15 | OP | 30.05 | 30.87 | 34.03 | 40 | 37.49 |
| | C | 28.2 | 29.37 | 31.58 | 40 | 40 |
| 16 | OP | 30.27 | 32.21 | 34.1 | 40 | 40 |
| | C | 35.49 | 36.39 | 39.08 | 40 | 33.69 |
| 17 | OP | 31.54 | 32.43 | 35.19 | 40 | 40 |
| | C | 32.04 | 33.08 | 36.1 | 40 | 40 |
| 18 | OP | 35.91 | 36.01 | 40 | 40 | 40 |
| | C | 36.34 | 40 | 40 | 40 | 30.25 |
| 19 | OP | 32.39 | 33.12 | 35.56 | 40 | 40 |
| | C | 29.08 | 29.94 | 32.94 | 40 | 40 |
| 20 | OP | 30.05 | 31.29 | 33.79 | 40 | 40 |
| | C | 30.92 | 31.5 | 34.09 | 40 | 40 |
| 21 | OP | 27.47 | 28.06 | 30.67 | 40 | 40 |
| | C | 29.14 | 29.98 | 31.8 | 40 | 40 |
| 22 | OP | 24.75 | 25.74 | 27.77 | 40 | 40 |
| | C | 23.09 | 24.34 | 26.48 | 40 | 40 |
| 23 | OP | 23.78 | 23.81 | 26.38 | 40 | 40 |
| | C | 30.05 | 30.82 | 32.61 | 40 | 40 |
| 24 | OP | 26.03 | 26.28 | 28.61 | 40 | 36.54 |
| | C | 20.33 | 19.15 | 21.83 | 40 | 33.46 |

## Slide 3
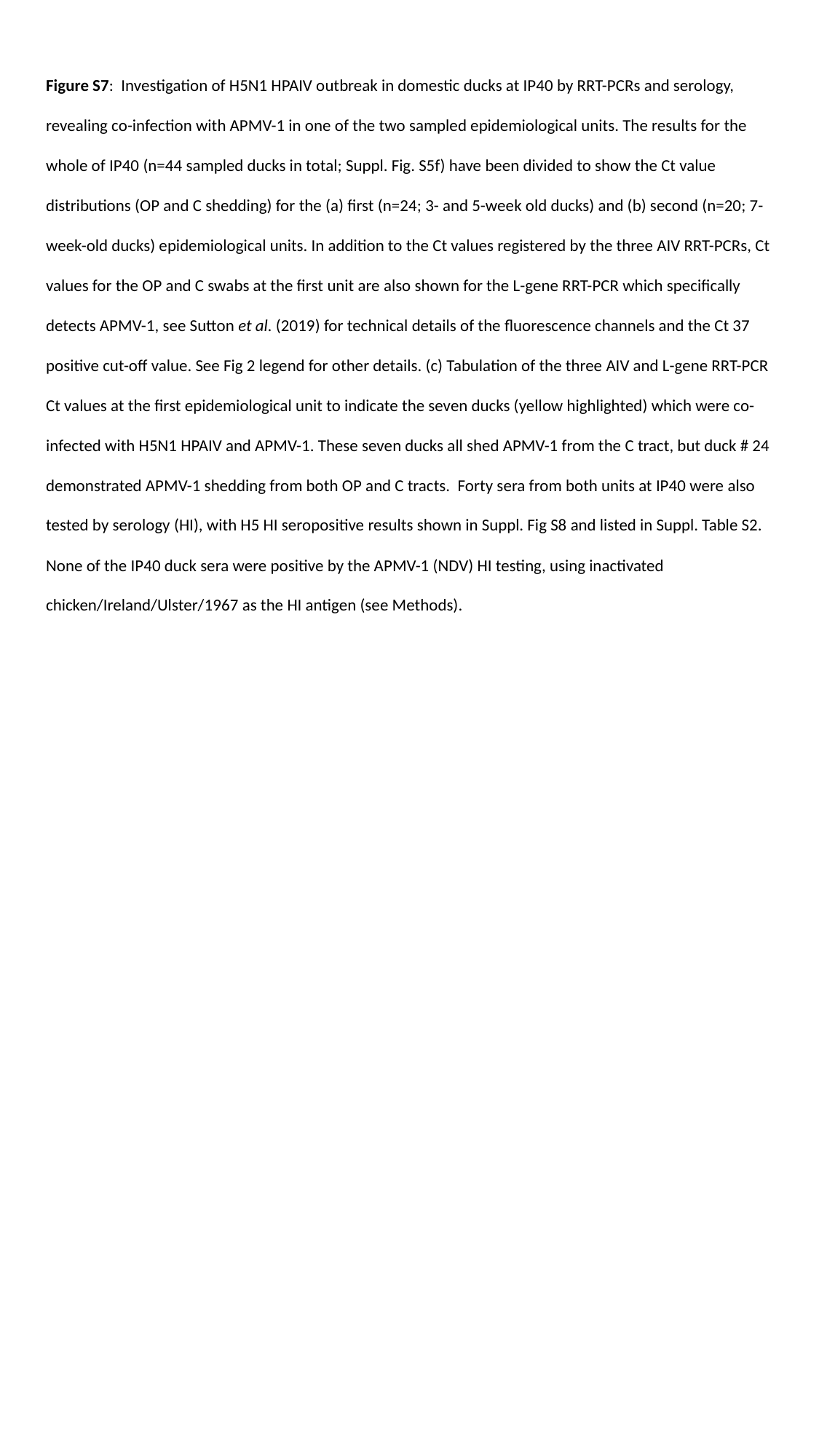

Figure S7: Investigation of H5N1 HPAIV outbreak in domestic ducks at IP40 by RRT-PCRs and serology, revealing co-infection with APMV-1 in one of the two sampled epidemiological units. The results for the whole of IP40 (n=44 sampled ducks in total; Suppl. Fig. S5f) have been divided to show the Ct value distributions (OP and C shedding) for the (a) first (n=24; 3- and 5-week old ducks) and (b) second (n=20; 7-week-old ducks) epidemiological units. In addition to the Ct values registered by the three AIV RRT-PCRs, Ct values for the OP and C swabs at the first unit are also shown for the L-gene RRT-PCR which specifically detects APMV-1, see Sutton et al. (2019) for technical details of the fluorescence channels and the Ct 37 positive cut-off value. See Fig 2 legend for other details. (c) Tabulation of the three AIV and L-gene RRT-PCR Ct values at the first epidemiological unit to indicate the seven ducks (yellow highlighted) which were co-infected with H5N1 HPAIV and APMV-1. These seven ducks all shed APMV-1 from the C tract, but duck # 24 demonstrated APMV-1 shedding from both OP and C tracts. Forty sera from both units at IP40 were also tested by serology (HI), with H5 HI seropositive results shown in Suppl. Fig S8 and listed in Suppl. Table S2. None of the IP40 duck sera were positive by the APMV-1 (NDV) HI testing, using inactivated chicken/Ireland/Ulster/1967 as the HI antigen (see Methods).
