# Supplementary material for: Efficient and Informative Laboratory Testing for Rapid Confirmation of H5N1 (Clade 2.3.4.4) High-Pathogenicity Avian Influenza Outbreaks in the United Kingdom
Source: Viruses. 2023 Jun 9;15(6):1344. doi: 10.3390/v15061344 (PMC10304448; doi:10.3390/v15061344)
Supplement: Supplementary file 1 [file viruses-15-01344-s001.zip › Fig S8.pptx]

## Slide 1
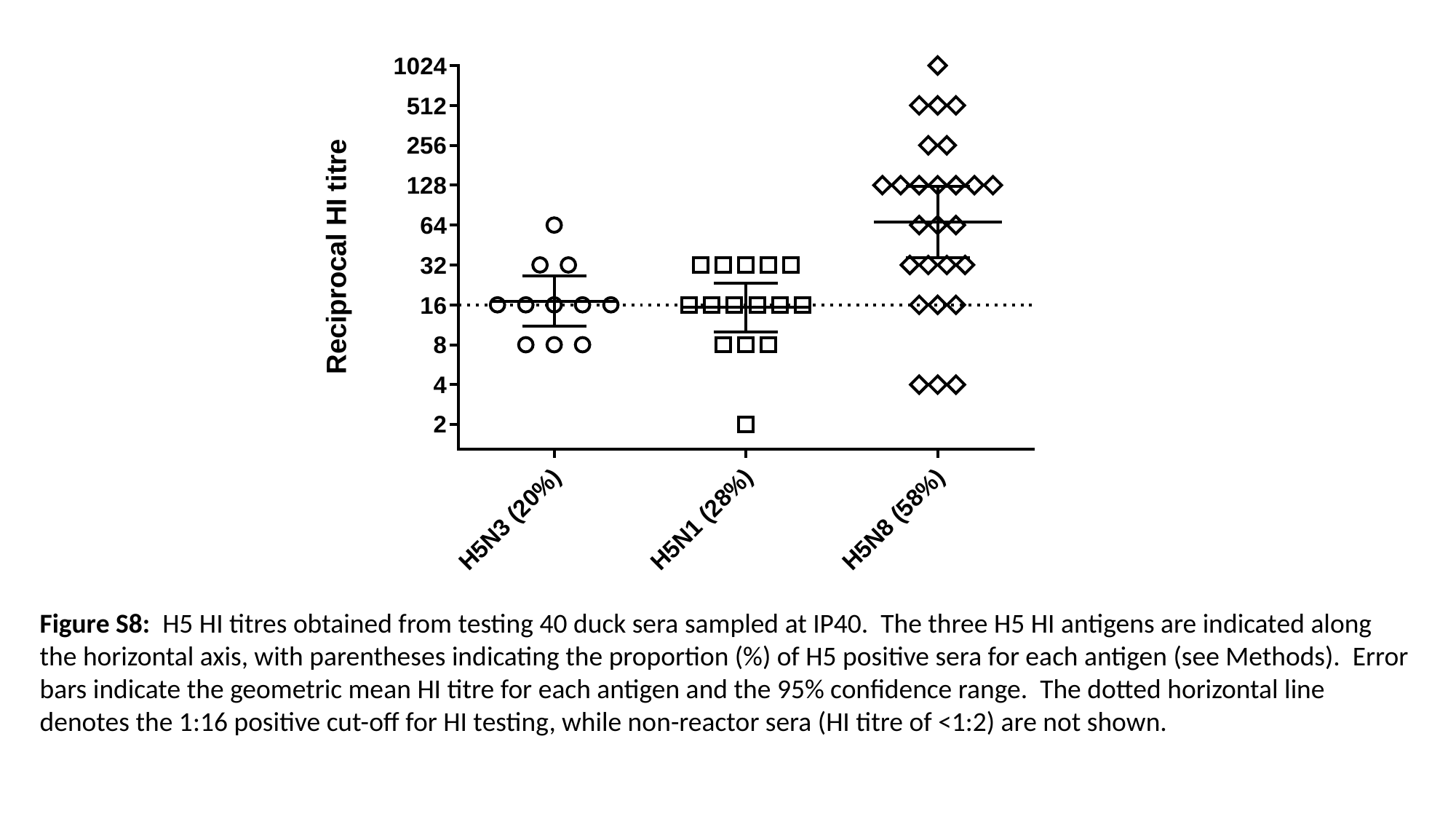

Figure S8: H5 HI titres obtained from testing 40 duck sera sampled at IP40. The three H5 HI antigens are indicated along the horizontal axis, with parentheses indicating the proportion (%) of H5 positive sera for each antigen (see Methods). Error bars indicate the geometric mean HI titre for each antigen and the 95% confidence range. The dotted horizontal line denotes the 1:16 positive cut-off for HI testing, while non-reactor sera (HI titre of <1:2) are not shown.
